# Early prediction of acute kidney injury biomarkers after endovascular stent graft repair of aortic aneurysm: a prospective observational study

**DOI:** 10.1186/s40560-014-0045-4

**Published:** 2014-07-31

**Authors:** Kazuyoshi Ueta, Michiko Watanabe, Naoya Iguchi, Akinori Uchiyama, Yukitoshi Shirakawa, Toru Kuratani, Yoshiki Sawa, Yuji Fujino

**Affiliations:** 1 Department of Anesthesiology and Intensive Care, Graduate School of Medicine, Osaka University, 2-15, Yamadaoka, Suita 565-0871, Japan; 2 Department of Cardiovascular Surgery, Graduate School of Medicine, Chiba University, 1-8-1, Inohana, Chuo-ku 260-8670, Chiba, Japan; 3 Department of Cardiovascular Surgery, Graduate School of Medicine, Osaka University, 2-15, Yamadaoka, Suita 565-0871, Japan

**Keywords:** Acute kidney injury, Neutrophil gelatinase-associated lipocalin, Biomarkers, Endovascular aortic repair

## Abstract

**Background:**

Acute kidney injury (AKI) is a common and serious condition usually detected some time after onset by changes in serum creatinine (sCr). Although stent grafting to repair aortic aneurysms is associated with AKI caused by surgical procedures or the use of contrast agents, early biomarkers for AKI have not been adequately examined in stent graft recipients. We studied biomarkers including urinary neutrophil gelatinase-associated lipocalin (NGAL), blood NGAL, *N*-acetyl-β-d-glucosaminidase (NAG), microalbumin (Alb), and liver fatty acid-binding protein (L-FABP) as prospective early biomarkers for AKI in patients who had received stent graft repairs of aortic aneurysms.

**Methods:**

In addition to pre-surgical sampling, at 2 to 6 h and at 1, 3 to 4, and 5 days or later (until stable) after surgery, urine and serum biomarkers were sampled from 47 patients who underwent stent graft repair of aortic aneurysms.

**Results:**

Using Acute Kidney Injury Network criteria, 6 (14%) of 42 retained patients developed AKI. NGAL corrected with urine Cr (NGAL/Cr) values demonstrated the best predictive value for AKI (97% specificity, 83% sensitivity at a 65.1 μg/gCr cutoff). The area under the receiver-operator characteristic curve of NGAL/Cr value 2 h after surgery was 0.9. Although NGAL/Cr, L-FABP corrected with urine Cr (L-FABP/Cr), L-FABP, NAG, and Alb corrected by urine Cr (Alb/Cr) all reached peak values before AKI detection by sCr in AKI patients, all biomarkers reached the cutoff value before AKI detection after adaption of cutoff value.

**Conclusions:**

After stent graft repair of aortic aneurysm, NGAL/Cr is a potentially useful early biomarker for AKI.

## Background

Acute kidney injury (AKI) after cardiac or other major surgery is associated with extended intensive care and prolonged hospital stay, diminished quality of life, shorter long-term survival, and other adverse effects [1]. Unfortunately, for a variety of reasons, serum creatinine (sCr) is a slow and unreliable indicator of AKI [2]. The failure of interventional trials to attenuate AKI has been attributed, in part, to late diagnosis. This has spurred recent interest in discovering and validating biomarkers that enable earlier diagnosis of AKI [2]. Initial studies have suggested promising candidates, including neutrophil gelatinase-associated lipocalin (NGAL), interleukin (IL)-18, liver fatty acid-binding protein (L-FABP), kidney injury molecule (KIM)-1 [2], and microalbumin (Alb) [3]. Krawczeski and colleagues [2] have reported, however, that when AKI occurs after cardiopulmonary bypass (CPB), urinary NGAL concentration rises more quickly than L-FABP, IL-18, and KIM-1.

Thoracic or abdominal aortic aneurysm is lethal if left untreated. Endovascular aortic repair with a stent graft is an alternative procedure that avoids open surgery [4]. Despite the risk of renal dysfunction owing to the surgical procedures themselves or to the use of contrast agents after stent graft surgery, we found few studies concerned with using novel renal biomarkers for the early detection of AKI [5],[6]. Although Chang and colleagues [5] reported increased blood NGAL (bNGAL) values in all patients after stent graft insertion, they did not measure urine biomarkers. In the other study, providing no useful evidence of the performance of novel AKI biomarkers, AKI after stent graft insertion occurred in only 1 of 34 patients [6].

Consequently, conjecturing that certain renal biomarkers are capable of providing earlier indication of AKI than sCr after stent graft surgery, we carried out experiments to test this hypothesis. We also evaluated the predictive ability of the tested biomarkers and how well they predicted subsequent long-term elevation of sCr. We wanted to find out whether some biomarkers were capable of early detection of AKI and subsequent long-term sCr elevation.

## Methods

### Patients

This study was approved by the Institutional Review Board of Osaka University Medical School. All patients undergoing endovascular stent graft repair of aortic aneurysm at our center from December 2010 to March 2011 were prospectively enrolled after obtaining written informed consent from each patient. Patients with pre-existing renal failure, indicated by sCr >3 mg/dL and those requiring renal replacement therapy were excluded.

### Study protocol

Considering the findings by Mishrav and colleagues [7], before surgery and after at 2 to 6 h and at 1, 3 to 4, and 5 days or more (until stable), 2 mL of blood and 10 mL of spot urine were collected from each patient. The blood and urine samples were spun at 2,000 × *g* within 24 h and the supernatant was frozen and stored at −80°C for later analysis. Urinary NGAL was evaluated using chemiluminescent immunoassay (CLIA; ARCHITECT®, Abbott Diagnostics, Abott Park, IL, USA), bNGAL using commercially available enzyme-linked immunosorbent assay (ELISA) kits (BioPorto Diagnostics, Grusbakken, Denmark), and urinary L-FABP using commercially available ELISA kits (CIMIC Co., Tokyo, Japan). Results for urinary *N*-acetyl-β-d-glucosaminidase (NAG), Alb, and creatinine were provided by a commercial lab. Values for sCr were obtained at baseline, 2 to 6 h after surgery and routinely monitored once a day. Besides measuring sCr and bNGAL, we tested biomarkers from urine. Results for urinary biomarkers such as NGAL, L-FABP, and albumin were normalized to urine creatinine concentrations and to compensate for possible urinary dilution or concentration, values are presented as urinary biomarker/Cr × 100.

AKI was characterized according to Acute Kidney Injury Network (AKIN) sCr criteria: stage 1, sCr rising to more than 0.3 mg/dL or to 50% over baseline; stage 2, increasing by 200% to 300%; stage 3, increasing by more than 300%. Because most patients returned to general wards after surgery, we did not include urine output criteria. Basic demographic, surgery duration, and hospital-stay data were collected. To evaluate the relationship of biomarkers and long-term outcome of kidney injury, sCr was recorded at 1, 3, and 6 months after surgery.

### Statistical analysis

Statistical analysis was performed using JMP version 9.0.3 (SAS Institute, Cary, NC, USA) and Analyse-it version 2.26 (Analyse-it Software, Leeds, West Yorkshire, UK).

Demographics, baseline measurements, and clinical outcomes of AKI and non-AKI patients were compared, as appropriate, using unpaired *t* testing, Pearson’s test, or Kruskal-Wallis test. Univariable logistic regression was used to assess the discriminative ability of biomarkers to predict AKI. Receiver-operator characteristic (ROC) curves were generated for each biomarker at 2 to 6 h after surgery. Cross comparison of areas under the curves (AUC) of each biomarker was done using methods developed by DeLong [8].

Data were expressed as mean ± SD (standard deviation), median (95% confidence interval) or median ± QD (quartile deviation) as appropriate and *P* < 0.05 was considered significant.

## Results

Five of 47 enrolled patients who underwent graft stent replacement therapy were excluded: 1 owing to incomplete data and 4 owing to urinary tract infection that caused continuously elevated levels of urine NGAL [9]. AKI occurred in 6 of the retained 42 patients and was detected 37 ± 44 h after surgery by sCr (Table 1). Five of these six AKI patients developed stage 1 AKI and one patient progressed to AKI stage 2. Each of the AKI group specifically underwent thoracic endovascular aortic repair (TEVAR, subtotal *N* = 18). None of the enrolled patients required dialysis or died within 30 days after surgery.

**Table 1:** Demographic data and clinical outcomes

	**Non-AKI**	**AKI**	***P*** **value**
*N*	36	6	
Operation type (TEVAR/EVAR)	18/18	6/0	0.007
Female (%)	14	0	0.33
Age (year)	70 ± 9	68 ± 13	0.65
Height (cm)	164 ± 7	169 ± 6	0.12
Weight (kg)	65 ± 11	72 ± 12	0.17
Diabetes mellitus (%)	25	16	0.64
Pre-operation sCr (mg/dL)	1.08 ± 0.27	0.95 ± 0.27	0.23
Surgery duration (min)	137 ± 53	231 ± 144	0.11
Hospital stay (day)	13.5 ± 11.8	22.5 ± 13.8	0.08

Data was expressed as mean ± SD except hospital stay (median ± quartile deviation). TEVAR, thoracic endovascular aortic repair; EVAR, endovascular aortic repair.

### Time-course elevation of biomarkers

**Figure 1:**
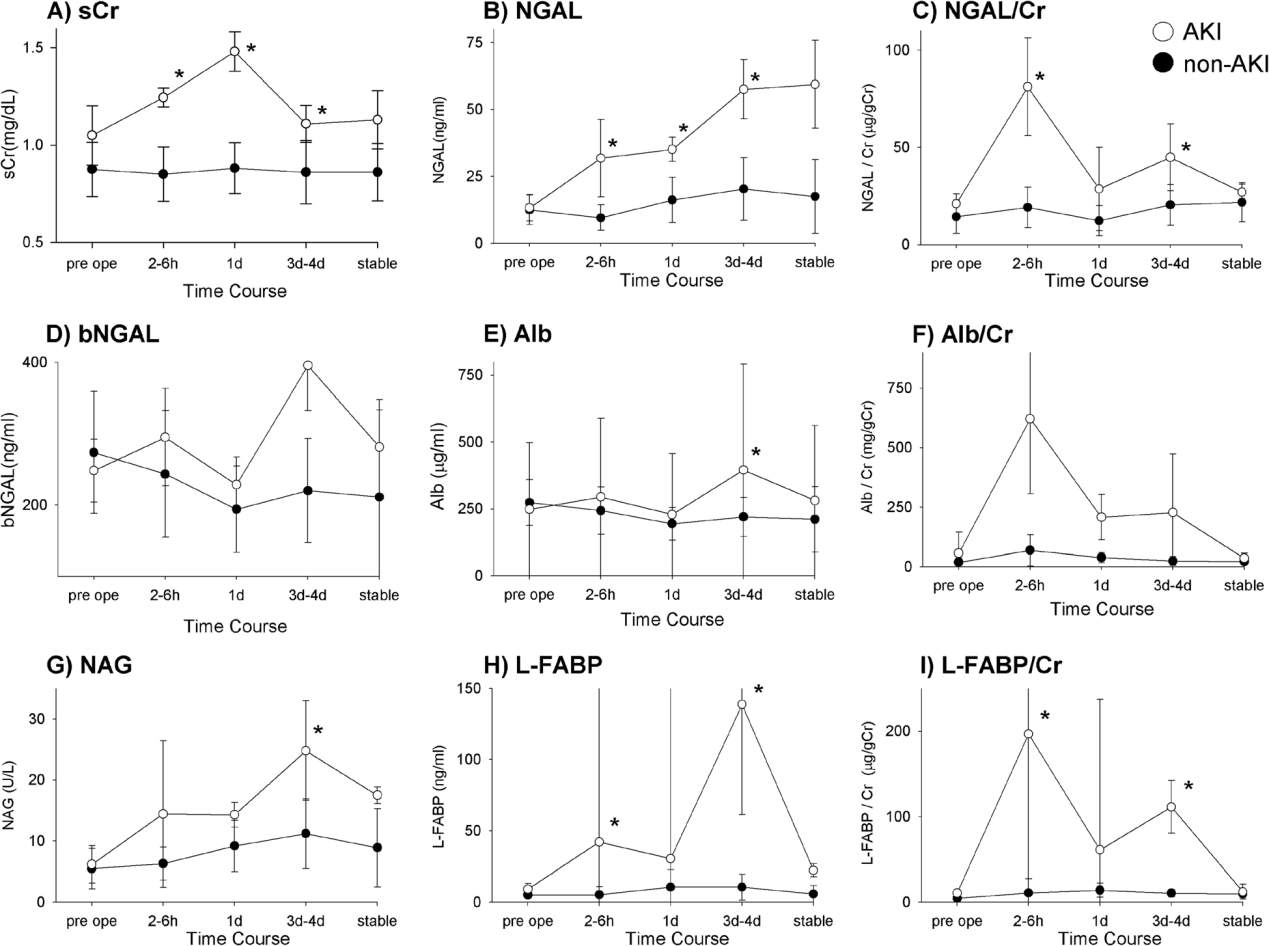
**Urinary biomarker concentration in patients with and without acute kidney injury.** Median and quartile deviations are shown. Asterisk indicates statistically significant differences (*P* < 0.05) in medians between patients with and without acute kidney injury. **A**, serum creatinine (sCr); **B**, urinary neutrophil gelatinase-associated lipocalin (NGAL); **C**, urinary NGAL corrected with urine creatinine (NGAL/Cr); **D**, blood NGAL (bNGAL); **E**, urinary microalbumin (Alb); **F**, Alb corrected with urine creatinine (Alb/Cr); **G**, *N*-acetyl-β-d-glucosaminidase (NAG) **H**, urinary liver fatty acid-binding protein (L-FABP) **I**, L-FABP corrected with urine creatinine (L-FABP/Cr).

As shown in Figure 1, during the sampling period, the values for all biomarkers from non-AKI patients remained consistently low. In AKI patients, values for each biomarker, except bNGAL and Alb/Cr, statistically significantly increased at some point after surgery. Statistically significant differences between sCr, NGAL, NGAL/Cr, L-FABP, and L-FABP/Cr values for AKI and non-AKI patients were first seen in samples taken at 2 to 6 h after surgery. Such differences for Alb and NAG were not apparent until postoperative day (POD) 3 or 4. Compared with the values, corrected with urine Cr (uCr) for each other biomarker in AKI patients, once risen, NGAL and L-FABP values remained elevated at subsequent measurements.

To assess if other biomarkers more quickly indicated AKI than sCr, the difference in time taken to reach peak value for each biomarker was compared with times to AKI detection by sCr (Table 2). Elapsed time after surgery was scaled as follows: 2 to 6 h, 4 h; more than 1 day, and POD (24-h units). NGAL/Cr, Alb/Cr, NAG, L-FABP, and L-FABP/Cr values peaked before sCr detection of AKI, and Alb peaked at about the same time as sCr detection.

**Table 2:** Time to reach maximum biomarker value, compared with serum creatinine detection of acute kidney injury

**Biomarker**	**Time (h)**
NGAL	29 (−32, 90)
NGAL/Cr	−32 (−85, 21)
bNGAL	20 (−31, 71)
Alb	0 (−55, 55)
Alb/Cr	−8 (−68, 52)
NAG	−7 (−61, 46)
L-FABP	−15 (−40, 10)
L-FABP/Cr	−29 (−79, 21)

Data was expressed as median (95% confidence interval). Minus sign indicates that the biomarker reached the peak value earlier than serum creatinine. NGAL, urinary neutrophil gelatinase-associated lipocalin; NGAL/Cr, urinary NGAL corrected with urine creatinine; bNGAL, blood neutrophil gelatinase-associated lipocalin; Alb, urinary microalbumin; Alb/Cr, urinary microalbumin corrected with urine creatinine; NAG, *N*-acetyl-β-d-glucosaminidase; L-FABP, urinary liver fatty acid-binding protein; L-FABP/Cr, urinary liver fatty acid-binding protein corrected with urine creatinine.

### Predictive ability of biomarkers

**Figure 2:**
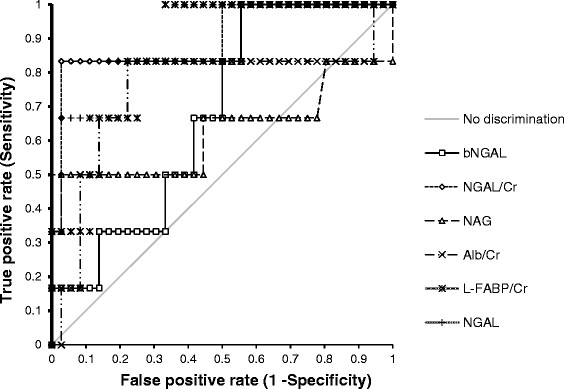
**Receiver-operator characteristic curves for biomarker prediction of acute kidney injury 2 to 6 h after surgery.** NGAL, urinary neutrophil gelatinase-associated lipocalin; NGAL/Cr, urinary NGAL corrected with urinary creatinine; bNGAL, blood NGAL; NAG, *N*-acetyl-β-d-glucosaminidase; Alb/Cr, urinary microalbumin corrected with urinary creatinine; L-FABP/Cr, urinary liver fatty acid-binding protein corrected with urine creatinine.

Figure 2 presents ROC curves derived from univariable logistic regression analysis for each biomarker value in samples taken 2 to 6 h after surgery. The ROC curve AUC for NGAL/Cr was 0.9, statistically significantly greater than for Alb/Cr (*P* = 0.04) (Table 3).

**Table 3:** Effectiveness of early biomarker prediction of AKI at 2 to 6 h after surgery: total patient population

**Biomarker**	**AUC**	**Cutoff**	**Sensitivity**	**Specificity**	**Diagnosis concordance**
NGAL	0.90	17.7 (ng/mL)	0.83	0.84	0.83
NGAL/Cr	0.90	65.1 (μg/gCr)	0.83	0.97	0.95
bNGAL	0.69	267.8 (ng/mL)	0.60	0.67	0.61
Alb	0.76	81.5 (μg/mL)	0.60	0.87	0.83
Alb/Cr	0.78	210 (mg/gCr)	0.80	0.90	0.89
NAG	0.62	6.6 (U/L)	0.66	0.57	0.59
L-FABP	0.87	25.1 (ng/mL)	0.83	0.83	0.83
L-FABP/Cr	0.86	36.7 (μg/gCr)	0.83	0.74	0.76
sCr	0.88	1.17 (mg/dL)	0.83	0.69	0.85

AUC, area under the curve; NGAL, urinary neutrophil gelatinase-associated lipocalin; NGAL/Cr, urinary NGAL corrected with urine creatinine; bNGAL, blood neutrophil gelatinase-associated lipocalin; Alb, urinary microalbumin; Alb/Cr, urinary microalbumin corrected with urine creatinine; NAG, *N*-acetyl-β-d-glucosaminidase; L-FABP, urinary liver fatty acid-binding protein; L-FABP/Cr, urinary liver fatty acid-binding protein corrected with urine creatinine; sCr, serum creatinine.

From points on the ROC curve, we determined the best cutoff values indicating maximum sensitivity and specificity (Table 3). From the tested biomarkers, NGAL/Cr performed the best, with sensitivity of 0.83 and specificity of 0.97. This result is almost same as for the TEVAR-only group because of the absence of AKI in the EVAR group (Table 4).

**Table 4:** Effectiveness of early biomarker prediction of AKI at 2 to 6 h after surgery: TEVAR group

**Biomarker**	**AUC**	**Cutoff**	**Sensitivity**	**Specificity**	**Diagnosis concordance**
NGAL	0.91	15.2 (ng/mL)	0.83	0.83	0.73
NGAL/Cr	0.90	65.1 (μg/gCr)	0.83	0.94	0.91
bNGAL	0.70	267.8 (ng/mL)	0.67	0.64	0.60
Alb	0.72	81.5 (μg/mL)	0.67	0.70	0.69
Alb/Cr	0.72	210 (mg/gCr)	0.83	0.78	0.78
NAG	0.59	6.6 (U/L)	0.67	0.47	0.48
L-FABP	0.82	25.1 (ng/mL)	0.83	0.71	0.73
L-FABP/Cr	0.79	36.8 (μg/gCr)	0.83	0.64	0.69
sCr	0.89	1.17 (mg/dL)	0.83	0.82	0.83

TEVAR, thoracic endovascular aortic repair, AUC, area under the curve; NGAL, urinary neutrophil gelatinase-associated lipocalin; NGAL/Cr, urinary NGAL corrected with urine creatinine; bNGAL, blood neutrophil gelatinase-associated lipocalin; Alb, urinary microalbumin; Alb/Cr, urinary microalbumin corrected with urine creatinine; NAG, *N*-acetyl-β-d-glucosaminidase; L-FABP, urinary liver fatty acid-binding protein; L-FABP/Cr, urinary liver fatty acid-binding protein corrected with urine creatinine; sCr, serum creatinine.

After detecting the cutoff values for each biomarker, we compared the difference in time taken to reach the cutoff value for each biomarker with the time to AKI detection by sCr (Table 5). All biomarkers reached cutoff values before sCr detection; NGAL/Cr, Alb/Cr, and L-FABP/Cr reached cutoff values much earlier than other biomarkers.

**Table 5:** **Time to reach cutoff value of each biomarker, compared with serum** cr**eatinine detection of AKI**

**Biomarker**	**Time (h)**
NGAL	−30 (−68, 8)
NGAL/Cr	−40 (−96, 16)
bNGAL	−19(−71, 33)
Alb	−20 (−51, −2)
Alb/Cr	−40 (−96, 16)
NAG	−19 (−37, 0)
L-FABP	−30 (−76, 16)
L-FABP/Cr	−40 (−96,16)

Data was expressed as median (95% confidence interval). Minus sign indicates that biomarker reached the cutoff value earlier than serum creatinine. NGAL, urinary neutrophil gelatinase-associated lipocalin; NGAL/Cr, urinary NGAL corrected with urine creatinine; bNGAL, blood neutrophil gelatinase-associated lipocalin; Alb, urinary microalbumin; Alb/Cr, urinary microalbumin corrected with urine creatinine; NAG, *N*-acetyl-β-d-glucosaminidase; L-FABP, urinary liver fatty acid-binding protein; L-FABP/Cr, urinary liver fatty acid-binding protein corrected with urine Cr.

### Ability to predict long-term sCr value elevation

Each biomarker was evaluated taking the maximum value or the 2 to 6 h postsurgical value. At 1, 3, and 6 months after surgery, none of the tested biomarkers was statistically significantly associated with higher levels of sCr than before surgery.

## Discussion

This study evaluated novel biomarkers for AKI after endovascular stent graft surgery. We found that NGAL corrected with uCr provided the earliest and most reliable indication of AKI. On the other hand, these early values did not predict longer-term sCr values.

As a promising alternative to open-surgery procedures, endovascular aortic repair is increasingly applied for aortic aneurysm and dissection [4]. The advantages of endovascular aneurysm repair endovascular aortic repair (EVAR) and TEVAR, including shorter operating time, less blood loss, and shorter hospital stay, have to be weighed against increased risk of renal dysfunction owing to the surgical procedures themselves or to the use of imaging contrast agents [4]. In the current study, the incidence of AKI was 14% after stent graft repair surgery, which is in line with previous reports (1.5% to 34%) [4], although it must be noted that AKI criteria vary according to study [10]. Because sCr, the conventional marker of renal function, is affected by several non-renal factors such as muscle mass, age, and sex, and does not usually peak until 1 to 3 days after cardiac surgery, alternative effective early diagnosis of AKI would facilitate more rapid therapeutic intervention and could help reduce the development of AKI [11].

With this end in view, several new biomarkers have been proposed and evaluated [12]. Measured on the day of AKI diagnosis, IL-18 and albumin values to creatinine ratio have been associated with higher risk of AKI progression and worse outcomes after cardiac surgery [13]. After adult cardiac surgery, L-FABP and NAG values have been shown to be useful indicators of AKI [14]. Meanwhile, it has been suggested that urine NGAL concentrations, which peak earliest during renal infection after cardiopulmonary bypass procedures, have the highest predictive value for AKI [2]. This early presence occurs owing to upregulation, just minutes after ischemia-reperfusion injury, of NGAL by renal distal tubular cells and its secretion into urine [15]. Corroborating Krawczeski’s findings, of the biomarkers we tested, we also importantly found that NGAL values at 2 to 6 h after surgery provided the best and most reliable prediction of AKI.

Possibly related to the abundance of NGAL/MMP-9 complexes associated with human aortic aneurysms [16], which may find their way into the bloodstream, Chang and colleagues [5] have reported increased bNGAL values in all patients after stent graft insertion. The extent of this association has yet to be determined, however, because both the ELISA kit (R&D Systems, Minneapolis, MN, USA) used in that study and the assay kit we used are unable to evaluate NGAL/MMP-9 complexes. Moreover, in medical conditions that stent graft patients are susceptible to, such as infection or diabetes mellitus, possibly due to the release of NGAL from cell types in organs other than kidney, in the present study, analysis of the bNGAL data that we acquired was unable to provide sufficiently reliable prediction of sCr detection of AKI after stent graft surgery. The duration of surgery was also shorter than in the report of Chang and colleagues. Consequently, in our samples, there may have been less bNGAL released in response to general systemic inflammation. In most clinical studies, urinary NGAL has been reported to be five to ten times higher than plasma NGAL after kidney injury and this may consequently make urinary NGAL more suitable for faster detection with AKI with greater specificity [11],[15],[16].

We were unable to find previous results for comparison of AKI after TEVAR and non-thoracic endovascular aneurysm repair but since all the patients in our AKI group underwent TEVAR, it seems likely that TEVAR increases the risk of renal ischemia [17] and microembolism [4]. There is also evidence that suprarenal stent-graft fixation is significantly more likely to increase urine microalbumin than infrarenal fixation [18].

Although all biomarkers reached cutoff values before sCr AKI detection, among adapted cutoff values in our small population, NGAL/Cr was the most reliable of the early indicators of AKI detected.

Four patients (9%) of the 47 originally enrolled also had to be excluded owing to urinary tract infection, because high NGAL values associated with such infection may have caused false positive detection of AKI [9]. Testing for a combination of biomarkers along with NGAL may improve the reliability of early detection of AKI in cardiovascular surgery patients with multiple complications, including urinary tract infection.

While AKI is commonly associated with prolonged hospital stay, greater healthcare costs, and earlier mortality [12], in the current study we found no statistically significant difference in the length of hospital stay. This may be because endovascular surgery is less invasive than open surgery and because our AKI group patient sCr values returned to baseline soon after surgery. Similarly, we found no association with any of our renal biomarker results and subsequent long-term detection of sCr elevation after surgery. Although our conclusions may be revised in light of the findings from further studies including, for example, sampling of larger populations or analysis of the effects of surgical procedures on the renal artery, after stent graft repair surgery, with the proviso that correction with urine Cr may be necessary because NGAL can detect AKI more quickly than earliest detection using sCr, we advocate testing for urinary NGAL, corrected by uCr, as an early diagnostic indicator of AKI.

### Limitations

This study has some obvious limitations.

Owing to lack of human resources, we were unable to fix specific time points for sampling and settled, for example, for 2 to 6 h and 3 to 4 days, which yields data that is not as precise as it could be. Our sampling-point decisions, however, were based on the findings of Mishra and colleagues, who reported that urinary NGAL peaks at 4 h and plateaus 60 h after surgery: they measured NGAL once a day after POD 1 in patients who had undergone cardiac surgery [7].

Although we expected a 20% to 25% incidence of AKI, the incidence was only 14%. Our smaller sample size may have introduced greater alpha error in the statistical analysis. Human resource limitations and experimental time constraints, however, did not allow further patient enrollment.

Meanwhile, for one patient in our AKI group, although clinical signs of renal failure such as low urine output and sCr elevation became apparent soon after surgery, bNGAL, NAG, and L-FABP were the only biomarker values that exceeded the relevant cutoff values. In some studies, NGAL has not performed well, resulting in false negative results [2],[19]. Consequently, the usefulness of NGAL as a biomarker for diagnosing AKI has yet to be established, and further study is obviously required to obtain more data for mortality, dialysis requirement, ICU stay, length of hospital stay, and other relevant outcomes.

## Conclusions

The results of this first study to evaluate the effectiveness of novel biomarkers for AKI after stent graft repair surgery have shown that NGAL corrected by uCr can provide early detection and prediction of AKI.

## Abbreviations

AKI: acute kidney injury

Alb: microalbumin

AUC: area under the receiver–operator characteristic curve

bNGAL: blood NGAL

CLIA: chemiluminescent immunoassay

CPB: cardiopulmonary bypass

ELISA: enzyme-linked immunosorbent assay

EVAR: endovascular aortic repair

IL-18: interleukin-18

KIM-1: kidney injury molecule-1

L-FABP: urinary liver fatty acid-binding protein

NAG: *N*-acetyl-β-d-glucosaminidase

NGAL: urinary neutrophil gelatinase-associated lipocalin

NGAL/Cr: urinary NGAL corrected with urine Cr

POD: postoperative day

QD: quartile deviation

ROC: receiver-operator characteristic

sCr: serum creatinine

SD: standard deviation

TEVAR: thoracic endovascular aortic repair

uCr: urine creatinine

## Competing interests

None of the authors have any conflicts of interest associated with this study.

## Authors’ contributions

KU is involved in the execution of the study, data analysis, and report writing. MW is responsible for the study design and data collection. NI is involved in study design and execution and data analysis. AU, YS, and YF are responsible for the study design and execution, data analysis, and report writing. TK and YS are also involved in the study design and execution and data analysis and provided advice during write up. All authors read and approved the final manuscript.

## References

[B1] Lok CE Austin PC Wang H Tu JV Impact of renal insufficiency on short- and long-term outcomes after cardiac surgery Am Heart J 2004 148 430 438 10.1016/j.ahj.2003.12.042 15389229

[B2] Krawczeski CD Goldstein SL Woo JG Wang Y Piyaphanee N Ma Q Bennett M Devarajan P Temporal relationship and predictive value of urinary acute kidney injury biomarkers after pediatric cardiopulmonary bypass J Am Coll Cardiol 2011 58 2301 2309 10.1016/j.jacc.2011.08.017 22093507 PMC3220882

[B3] Yu Y Jin H Holder D Ozer JS Villarreal S Shughrue P Shi S Figueroa DJ Clouse H Su M Muniappa N Troth SP Bailey W Seng J Aslamkhan AG Thudium D Sistare FD Gerhold DL Urinary biomarkers trefoil factor 3 and albumin enable early detection of kidney tubular injury Nat Biotechnol 2010 28 470 477 10.1038/nbt.1624 20458317

[B4] Pisimisis GT Khoynezhad A Bashir K Kruse MJ Donayre CE White RA Incidence and risk factors of renal dysfunction after thoracic endovascular aortic repair J Thorac Cardiovasc Surg 2010 140 S161 S167 10.1016/j.jtcvs.2010.10.014 21092786

[B5] Chang CK Chuter TA Niemann CU Shlipak MG Cohen MJ Reilly LM Hiramoto JS Systemic inflammation, coagulopathy, and acute renal insufficiency following endovascular thoracoabdominal aortic aneurysm repair J Vasc Surg 2009 49 1140 1146 10.1016/j.jvs.2008.11.102 19394543 PMC3276362

[B6] Brulotte V Leblond FA Elkouri S Therasse E Pichette V Beaulieu P Bicarbonates for the prevention of postoperative renal failure in endovascular aortic aneurysm repair: a randomized pilot trial Anesthesiol Res Pract 2013 2013 467326 23840204 10.1155/2013/467326 PMC3694372

[B7] Mishra J Dent C Tarabishi R Mitsnefes MM Ma Q Kelly C Ruff SM Zahedi K Shao M Bean J Mori K Barasch J Devarajan P Neutrophil gelatinase-associated lipocalin (NGAL) as a biomarker for acute renal injury after cardiac surgery Lancet 2005 365 1231 1238 10.1016/S0140-6736(05)74811-X 15811456

[B8] DeLong ER DeLong DM Clarke-Pearson DL Comparing the areas under two or more correlated receiver operating characteristic curves: a nonparametric approach Biometrics 1988 44 837 845 10.2307/2531595 3203132

[B9] Yilmaz A Sevketoglu E Gedikbasi A Karyagar S Kiyak A Mulazimoglu M Aydogan G Ozpacaci T Hatipoglu S Early prediction of urinary tract infection with urinary neutrophil gelatinase associated lipocalin Pediatr Nephrol 2009 24 2387 2392 10.1007/s00467-009-1279-6 19649660

[B10] Mehta RL Pascual MT Gruta CG Zhuang S Chertow GM Refining predictive models in critically ill patients with acute renal failure J Am Soc Nephrol 2002 13 1350 1357 10.1097/01.ASN.0000014692.19351.52 11961023

[B11] Parikh CR Devarajan P Zappitelli M Sint K Thiessen-Philbrook H Li S Kim RW Koyner JL Coca SG Edelstein CL Shlipak MG Garg AX Krawczeski CD Postoperative biomarkers predict acute kidney injury and poor outcomes after pediatric cardiac surgery J Am Soc Nephrol 2011 22 1737 1747 10.1681/ASN.2010111163 21836147 PMC3171944

[B12] Soni SS Ronco C Katz N Cruz DN Early diagnosis of acute kidney injury: the promise of novel biomarkers Blood Purif 2009 28 165 174 10.1159/000227785 19590184

[B13] Koyner JL Garg AX Coca SG Sint K Thiessen-Philbrook H Patel UD Shlipak MG Parikh CR Biomarkers predict progression of acute kidney injury after cardiac surgery J Am Soc Nephrol 2012 23 905 914 10.1681/ASN.2011090907 22383693 PMC3338298

[B14] Katagiri D Doi K Honda K Negishi K Fujita T Hisagi M Ono M Matsubara T Yahagi N Iwagami M Ohtake T Kobayashi S Sugaya T Noiri E Combination of two urinary biomarkers predicts acute kidney injury after adult cardiac surgery Ann Thorac Surg 2012 93 577 583 10.1016/j.athoracsur.2011.10.048 22269724

[B15] Wagener G Minhaz M Mattis FA Kim M Emond JC Lee HT Urinary neutrophil gelatinase-associated lipocalin as a marker of acute kidney injury after orthotopic liver transplantation Nephrol Dial Transplant 2011 26 1717 1723 10.1093/ndt/gfq770 21257679 PMC3145384

[B16] Perry TE Muehlschlegel JD Liu KY Fox AA Collard CD Shernan SK Body SC Plasma neutrophil gelatinase-associated lipocalin and acute postoperative kidney injury in adult cardiac surgical patients Anesth Analg 2010 110 1541 1547 10.1213/ANE.0b013e3181da938e 20435938 PMC2999841

[B17] Miller CC 3rd Grimm JC Estrera AL Azizzadeh A Coogan SM Walkes JC Safi HJ Postoperative renal function preservation with nonischemic femoral arterial cannulation for thoracoabdominal aortic repair J Vasc Surg 2010 51 38 42 10.1016/j.jvs.2009.08.044 19853401 PMC2815229

[B18] Kouvelos GN Boletis I Papa N Kallinteri A Peroulis M Matsagkas MI Analysis of effects of fixation type on renal function after endovascular aneurysm repair J Endovasc Ther 2013 20 334 344 10.1583/12-4177MR.1 23731306

[B19] Haase M Bellomo R Devarajan P Schlattmann P Haase-Fielitz A Accuracy of neutrophil gelatinase-associated lipocalin (NGAL) in diagnosis and prognosis in acute kidney injury: a systematic review and meta-analysis Am J Kidney Dis 2009 54 1012 1024 10.1053/j.ajkd.2009.07.020 19850388

